# PP59 Supporting The Brazilian Regulatory Agency For Supplementary Healthcare: A Project From The NATS/NEv - Hospital Sírio-Libanês

**DOI:** 10.1017/S026646232510278X

**Published:** 2025-12-29

**Authors:** Carolina de Oliveira Cruz Latorraca, Patrícia do Carmo Silva Parreira, Rafael Leite Pacheco, Ana Luiza Cabrera Martimbianco, Verônica Colpani, Isabela Porto de Toledo, Roberta Borges Silva, Camila Monteiro Cruz, Cecília Menezes Farinasso, Rachel Riera

## Abstract

**Introduction:**

The rapid evolution of health technologies presents opportunities and challenges for the integration of new procedures and treatments within health systems. In Brazil, the National Supplementary Health Agency (ANS) plays a key role in ensuring the continuous updating of their catalog of drugs, devices, and procedures, which defines the coverage of health services in the supplementary sector. This study detailed an initiative designed to provide ANS with evidence-based technical support.

**Methods:**

The Hospital Sírio-Libanês, in collaboration with the ANS and the Ministry of Health, has supported health technology assessment strategies that facilitate updating the catalog of drugs, devices, and procedures of the ANS in the “Supporting the Brazilian regulatory agency for supplementary healthcare through health technology assessment actions” project. Initiated in 2019, the project is now entering its third three-year phase (2024 to 2026).

**Results:**

From 2019 to 2024, the project developed 97 technical-scientific health technology assessment reports or workshops, with 93 analyses of social participation and workshops. A total of 43 tutoring sessions on clinical and methodological topics were held for the ANS team involved in the catalog update, which were delivered through synchronous and asynchronous formats. The project developed five methodological templates and conducted three educational initiatives that trained approximately 100 professionals from ANS and the Ministry of Health in health technology assessment through remote synchronous courses. Furthermore, 17 scientific dissemination strategies were executed, including articles and presentations at events, which helped broaden knowledge and foster informed discussions on priority issues that can affect society.

**Conclusions:**

By encouraging evidence-based methods, this project has enhanced the ANS team’s decision-making capacity regarding coverage of the catalog of drugs, devices, and procedures of the ANS. This has supported the updating process, guaranteeing that decisions on technology incorporation are informed by the best scientific evidence available and consider effects on Unified Health System resources, benefiting health plan users and public health policies.

